# Investigation of Normally-Off p-GaN/AlGaN/GaN HEMTs Using a Self-Terminating Etching Technique with Multi-Finger Architecture Modulation for High Power Application

**DOI:** 10.3390/mi12040432

**Published:** 2021-04-14

**Authors:** Ya-Chun Chang, Yu-Li Ho, Tz-Yan Huang, Ding-Wei Huang, Chao-Hsin Wu

**Affiliations:** 1 Graduate Institute of Photonics and Optoelectronics, National Taiwan University, Taipei 106319, Taiwan; r08941054@ntu.edu.tw (Y.-C.C.); r07941088@ntu.edu.tw (Y.-L.H.); r09941004@ntu.edu.tw (T.-Y.H.); dwhuang@ntu.edu.tw (D.-W.H.); 2 Graduate Institute of Electronics Engineering, National Taiwan University, Taipei 106319, Taiwan

**Keywords:** gallium nitride (GaN), enhancement-mode, p-GaN HEMT, multi-finger layout, self-terminating etching

## Abstract

Normally-off p-gallium nitride (GaN) high electron mobility transistor (HEMT) devices with multi-finger layout were successfully fabricated by use of a self-terminating etching technique with Cl_2_/BCl_3_/SF_6_-mixed gas plasma. This etching technique features accurate etching depth control and low surface plasma damage. Several devices with different gate widths and number of fingers were fabricated to investigate the effect on output current density. We then realized a high current enhancement-mode p-GaN HEMT device with a total gate width of 60 mm that exhibits a threshold voltage of 2.2 V and high drain current of 6.7 A.

## 1. Introduction

Due to the advantages of gallium nitride (GaN) over silicon, GaN-based power devices have recently received widespread attention in power electronics applications as these devices exhibit high breakdown voltage, low on-resistance (*R_on_*), and fast switching speed [1,2,3,4,5]. The dominant platform for developing commercial GaN power electronic devices is based on lateral heterojunctions (e.g., AlGaN/GaN) grown on large-size, low cost silicon substrates [3].

However, the high-density two-dimensional electron gas (2DEG) induced by the strong polarization effect makes GaN high electron mobility transistors (HEMTs) exhibit normally-on behavior which increases the complexity of circuit design and introduce safety concerns. Enhancement-mode (E-mode) HEMTs with a positive threshold voltage (V_TH_) are more desirable for practical power switching applications [6,7,8].

In recent years, normally-off GaN HEMTs have been realized by several approaches such as fluorine plasma ion implantation [9], ultra-thin AlGaN barrier [10], recessed gate [11], and p-GaN gate [12]. Among them, the p-GaN gate HEMTs are the most promising solution owing to the stronger control over the gate region, superior R_on_ × Q_G_ (gate charge) figure of merit [13], and thermal stability, and have been recently commercialized in the power electronics market [14]. The working principle behind this design is that the conduction band under the gate is lifted up through the p-GaN cap, resulting in a normally-off operation with a positive threshold voltage.

For application in real power integrated circuits, the devices are required to have high current and high breakdown voltage capability [15], which are realized by increasing the total gate width and thus the device area. The critical issue of large-area devices is low yield [16,17]. Several methods have been reported to optimize large current device fabrication. Optimizing the Mg profile in the p-GaN layer and controlling the epitaxial growth condition are the most standard methods to improve device characteristics [18]. Devices with better dielectric quality have also been realized to achieve low leakage and low on-resistance [19]. Using thicker Au-plated ohmic electrodes has also been shown to increase the drain current [15]. Modifying the device geometry through variation of gate width or number of gate fingers can also effectively provide higher dissipated power capability when designing with a multi-finger layout [20].

However, few papers realize E-mode high drain current GaN HEMT devices through the actual fabrication process because of the challenging etching process. The two major challenges of p-GaN gate HEMTs are accurate etching uniformity control of the non-gated channel region [21,22,23] and plasma-induced damage on the underlying AlGaN surface during the p-GaN removal process [24,25]. The residual p-GaN layer will deplete the 2DEG density resulting in a decrease in current density. Likewise, over-etching of the AlGaN barrier layer will also decrease the current density due to decreasing the polarization effect [26]. Both conditions will deteriorate the conduction of the device. Thus, in order to maintain the 2DEG for low conduction resistance, etching of the p-GaN layer should stop on top of the AlGaN layer [27].

Traditionally, the p-GaN etching step makes use of Cl_2_/BCl_3_-mixed gas plasma in slow rate inductively coupled plasma reactive ion etching (ICP-RIE) [28]. The critical issue is that the slow etching rate is sensitive to the ICP chamber conditions. Therefore, it is difficult to have a stable and repeatable etching process because of the narrow window for etching time.

In this work, a p-GaN gate enhancement-mode GaN HEMT using a multi-finger layout was successfully demonstrated to achieve high current density by using Cl_2_/BCl_3_/SF_6_-based ICP etching along with endpoint detection (EPD) to have real-time monitoring of the etching depth. This technique features self-termination at an AlGaN barrier surface with a wider tolerance of etching time and etching uniformity. Furthermore, several devices with different gate widths and number of fingers were fabricated to investigate the effects on output current density. The realized E-mode GaN HEMT devices were characterized by DC measurements. For a device with a total gate width of 60 mm, the threshold voltage (V_TH_) is 2.2 V, and the drain current reaches 6.7 A, indicating a drain current density of 112.5 mA/mm.

## 2. Materials and Methods

**Figure 1:**
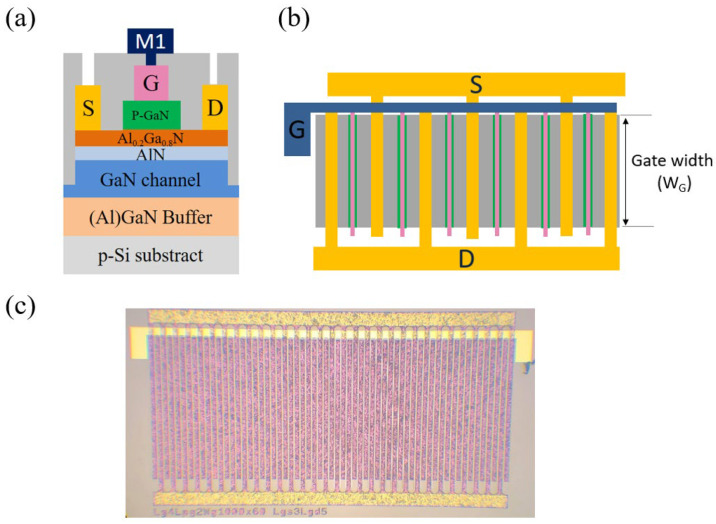
(**a**) Cross section of the p-gallium nitride (GaN) high electron mobility transistors (HEMT) device structure. (**b**) Schematic top view of the p-GaN HEMT device structure. (**c**) Optical micrograph of a realized p-GaN gate HEMT (individual gate width W_G_/# of fingers = 1000 μm/60, L_G_ = 4 μm, L_GD_ = 3 μm, and L_GS_ = 3 μm).

Figure 1a,b shows the cross-section of the p-GaN gate HEMTs and schematic top view of the p-GaN HEMT with multi-finger structure, respectively. The AlGaN/GaN heterostructures were grown by MOCVD on 800 μm p-Si substrates. The layer stack consisted of a 3.8 μm thick (Al)GaN buffer layer to enable high voltage operation, a 300 nm thick GaN channel layer, an 8 nm AlN spacer layer to effectively suppress alloy disorder scattering [29], and a 15 nm Al_0.2_Ga_0.8_N barrier layer. The top layer consisted of a 70 nm thick Mg-doped p-GaN layer with a doping concentration of 4 × 10^19^ cm^−3^.

**Figure 2:**
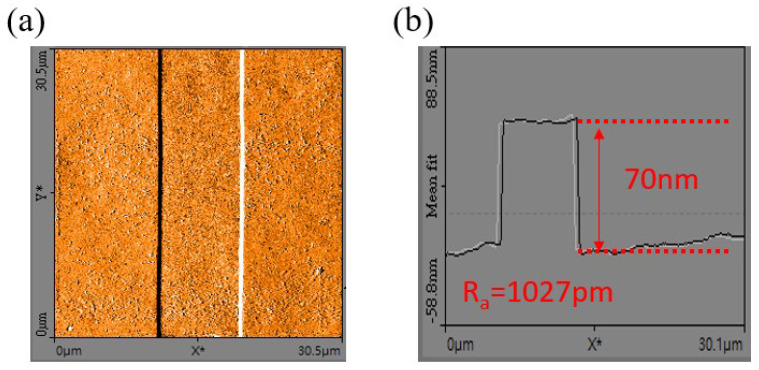
(**a**) Atomic force microscopy (AFM) image of the p-GaN gate region. (**b**) The depth profile of the p-GaN gate with an etching depth 70 nm and average roughness (*R_a_*) of 1027 pm.

The device fabrication started with active region isolation by mesa etching to a 200-nm depth using Cl_2_/BCl_3_ SAMCO ICP RIE-200iPC (inductively coupled plasma reactive ion etching). Then, the 7-μm long p-GaN gate region was protected using photoresist, and a high-selectivity Cl_2_/BCl_3_/SF_6_-mixed gas plasma etch was performed on the non-gated active region by using ICP RIE200i. In principle, when the SF_6_ plasma reaches the AlGaN barrier surface, the fluorine ion reacts with the Al atoms and forms a thin AlF_3_ etching stop layer (SF_6_ plasma + Al→AlF_3_). During the dry etching process, the employment of endpoint detection provides real-time monitoring of the etching depth where specific wavelengths of light (300–350 nm) are irradiated on the surface of the non-gated active region. After the light source reaches the surface, a portion of light is reflected directly from the surface, but some enters the wafer and is reflected back from the channel layer. Thus, the reflected light received by the detector is a combination of signals from each layer within the sample, and specific interference fringes are then formed and can be displayed on a monitor. If the etching depth does not change, the reflected light intensity would stay constant. A mixture of Cl_2_/BCl_3_/SF_6_ gas plasma was applied to remove the p-GaN cap for 132.8 s, and the reflected light intensity remained constant after that time indicating the end of the etching process. After that, the thin AlF_3_ layer on the surface was removed by a buffered oxide etchant (BOE) wet treatment for 1 min. The resulting surface and actual etching depth were measured by NanoSurf Flex atomic force microscopy (AFM) as shown in Figure 2. The etching depth was exactly 70 nm, the thickness of the p-GaN layer, and the average roughness (*R_a_*) was 1027 pm (30 × 30 μm^2^). Afterwards, Ti/Al/Ni/Au (25/125/40/150 nm) were used to form ohmic contacts as source and drain electrodes, followed by annealing in N_2_ ambient at 875 °C for 45 s using Premtek RTP-T41M (rapid thermal processing). Using a transmission line measurement (TLM), the channel sheet resistance and specific contact resistivity were 310 Ω/sq and 9469 Ω·μm^2^, respectively. The good ohmic contact and sheet resistance were due to the accurate etching depth (which maintains a high 2DEG density) and smooth surface with negligible ion bombardment damage. Ni/Au (15/280 nm) gate metal was deposited by e-beam evaporation to form a Schottky contact. Next, 300 nm thick SiNx surface passivation was deposited using Samco PD-220N PECVD to reduce the N vacancies on the device’s surface. Finally, after contact window opening on the gate regions, a thick Ti/Au (15/1300 nm) Metal 1 was deposited to serve as the gate electrode bridge. The realized large-area p-GaN HEMT device with multi-finger structure is shown in Figure 1c. The power device has a gate length (L_G_) of 4 μm, gate–source distance (L_GS_) of 3 μm, gate–drain distance (L_GD_) of 3 μm, and total gate width of 60 mm. The device DC characteristics were analyzed using an Agilent B1505A power device analyzer.

## 3. Results and Discussion 

In order to investigate the relationship between the output current density and multi-finger layout, p-GaN gate HEMT devices with different gate width (W_G_) and different number of fingers were fabricated simultaneously on the same chip. In Section 3.1, devices with single finger but different W_G_ are compared. In Section 3.2, devices with W_G_ = 60 μm but different number of fingers are also compared. In Section 3.3, a summary for these different layouts is discussed. Finally, in Section 3.4, a high drain current p-GaN HEMT with a multi-finger layout is realized and presented. 

### 3.1. Single Finger Devices with Different W_G_

**Figure 3:**
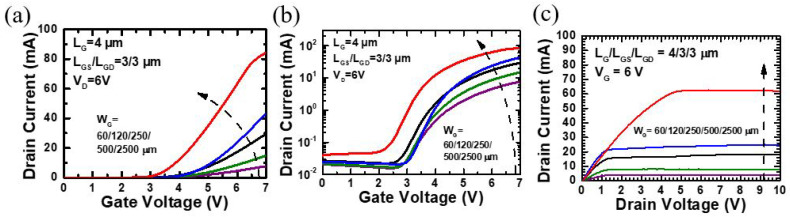
Device performance of single finger p-GaN HEMTs with different gate width (60/120/250/500/2500 μm) at V_DS_ = 6 V: (**a**) I_DS_-V_GS_ in linear scale, (**b**) I_DS_-V_GS_ in log scale, (**c**) and output characteristics.

As shown in Table 1, five single-finger devices with different gate width are labeled as *A* (W_G_ = 60 μm), *B* (W_G_ = 120 μm), *C* (W_G_ = 250 μm), *D* (W_G_ = 500 μm), and *E* (W_G_ = 2500 μm), while all other parameters (L_G_/L_GS_/L_GD_ = 4/3/3 μm) are held constant. Figure 3a,b shows the transfer curves of different W_G_ p-GaN HEMTs at V_DS_ = 6 V and V_GS_ = 0~7 V, and Figure 3c shows output performance at V_GS_ = 6 V and V_DS_ = 0~10 V.

As seen in Figure 3a,b, the drain current reaches 83.5 mA for device E (W_G_ = 2500 μm) and drops to 7.4 mA for device A (W_G_ = 60 μm). This result is consistent with the standard trend of Si-MOSFETs where the total current increases with longer gate width. However, as shown in Table 1, the current density of device A is four times greater than the current density of device E. That is to say, the current density decreases when the gate width increases. Meanwhile, according to Figure 3c, the on-resistance also dramatically drops when the gate width increases to 2500 μm. A possible reason for this tendency is that when the gate voltage is applied on the top finger region (blue gate in Figure 1b), the total voltage source cannot bias to the end of the individual gate fingers (Figure 1b green and pink gates) due to the long gate width. Thus, the gate cannot control the channel under the end of the gate finger, resulting in the 2DEG being unable to form. Table 1 summarizes the design parameters and electrical characteristics for devices with a single finger but different gate widths.

### 3.2. W_G_ = 60 μm Devices with Different Number of Fingers

As presented in Table 2, four devices with the same 60 μm gate width and different number of fingers are labeled as F (4 fingers), G (10 fingers), H (40 fingers), and I (60 fingers) while all other parameters (L_G_/L_GS_/L_GD_ = 4/3/3 μm) are held constant.

**Figure 4:**
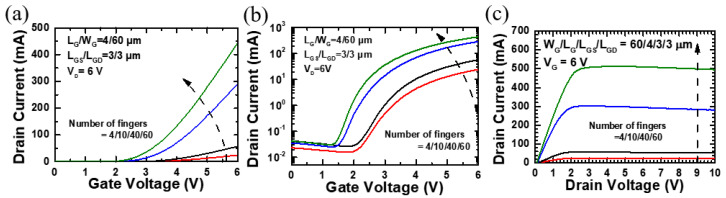
Device performance of p-GaN HEMTs (W_G_ = 60 μm) with different number of fingers (4/10/40/60) at V_DS_ = 6 V: (**a**) I_DS_-V_GS_ in linear scale, (**b**) I_DS_-V_GS_ in log scale, (**c**) and output characteristics.

Figure 4a,b depicts the I_DS_-V_GS_ transfer curves of the W_G_ = 60 μm devices with a different number of fingers at V_DS_ = 6 V. The output drain current at a drain bias of 6 V is 442 mA for device I (60 fingers) and 24 mA for device F (4 fingers). As shown in Table 2, the current density also increases with the number of fingers. This elevated current is due to the superposition of current from each finger. Moreover, according to Figure 4c, the rising output current results from the decrease of on-resistance from 45.68 Ω for device F (4 fingers) to 3.65 Ω for device I (60 fingers). Table 2 summarizes the design parameters and electrical characteristics for the devices with W_G_ = 60 μm but different number of fingers.

### 3.3. Summary of the Multi-Finger Layout Devices

**Figure 5:**
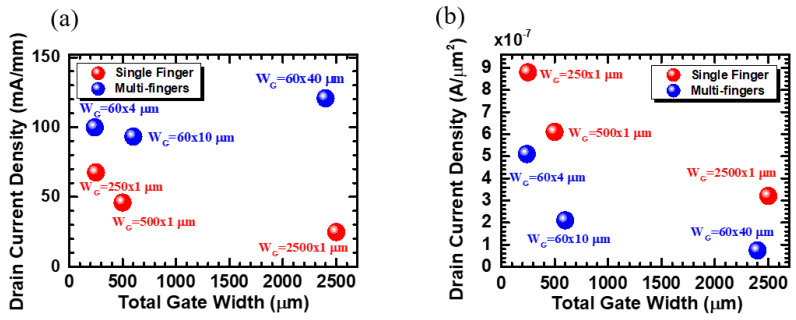
Drain current densities of p-GaN HEMTs with different total gate width. (**a**) Drain current density (normalized by W_G_) versus total gate width. (**b**) Drain current density (normalized by active area) versus total gate width.

In order to compare whether the modulation of gate width or number of fingers has a greater impact on the current density, the drain current density (mA/mm) is plotted against the total gate width in Figure 5a. Thus, devices with similar total gate width are more readily compared. For example, device C and device F which have total gate widths of 250 mm and 240 mm, respectively, are compared to show that when the devices have similar total gate width, the devices with a multi-finger structure (blue) have significantly higher current density than devices with a single finger layout (red). The current is greatly increased as the total gate width is close to 2500 μm. The results are consistent with the current commercial trend which commonly applies the multi-finger structure on the devices. The drain current per active area (A/μm^2^) is also plotted against the total gate width, as shown in Figure 5b. The results indicate that, with a similar total gate width, the devices with a multi-finger layout (blue) have higher output current density than the devices with a single finger structure (red). This result is attributed to the thermal crosstalk between individual gate fingers which may increase device temperature and also reduce the power density [20]. Meanwhile, a larger active area brings about more heat dissipation. Thus, increasing the active area of the multi-finger devices will likely improve the drain current per active area.

### 3.4. Large-Area p-GaN HEMT with High Drain Current Power Device Performance

**Figure 6:**
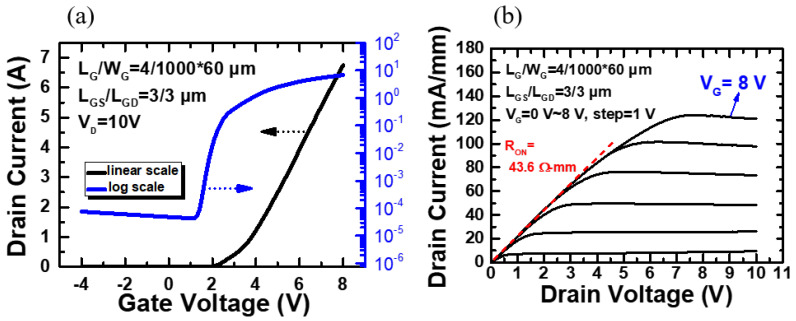
Device performance of the large-area p-GaN HEMT with total gate width 60 mm (1000 mm × 60 fingers). (**a**) Total drain current versus gate voltage at V_DS_ = 10 V in linear and log scale. (**b**) Output performance at V_DS_ = 0–10 V and V_GS_ = 0–8 V.

Based on these experimental results, a high current normally-off p-GaN HEMT device was fabricated. The device is designed with a total gate width of 60 mm (W_G_ = 1000 μm, number of fingers = 60), L_G_ of 4 μm, L_GD_ of 3 μm, and L_GS_ of 3 μm. The device DC characteristics are analyzed using an Agilent B1505A power device analyzer. The transfer curves of the devices in linear and log scale are shown in Figure 6a at V_DS_ = 10 V, and the output performance as a function of V_GS_ is presented in Figure 6b. 

The threshold voltage (V_TH_) is 2.2 V (defined by I_DS_ = 1 mA/mm), the subthreshold swing (SS) is 221.1 mV/dec, and the on/off ratio is 1.4 × 10^5^ which exhibits good switching characteristics. The output drain current and current density is 6.7 A and 112.5 mA/mm, respectively, at V_GS_ = 8 V and V_DS_ = 10 V, and the on-resistance (R_on_) is 43.6 Ω-mm at V_GS_ = 8 V.

## 4. Conclusions

In this work, a high current normally-off p-GaN HEMT device with multi-finger layout was successfully fabricated using a self-terminating etching technique with Cl_2_/BCl_3_/SF_6_-mixed gas plasma. Several devices with different gate width and number of fingers were fabricated to investigate the effects on output current density. The drain current reaches 83.5 mA for devices with W_G_ = 60 μm and drops to 7.4 mA for devices with W_G_ = 2500 μm. The decrease in current for long gate widths is due to the fact that the applied gate voltage on the top of the gate finger cannot bias to the end of the finger, resulting in the 2DEG being unable to form. Through modulating the number of fingers, the output drain current is 442 mA for devices with 60 fingers while only 24 mA for devices with four fingers. This elevated current is due to the superposition of current from each finger. Lastly, a high current normally-off W_G_ = 60 mm p-GaN HEMT device was realized with a threshold voltage of 2.2 V and drain current of 6.7 A.

## Figures and Tables

**Table 1:** Design parameters for single finger devices with different gate widths (W_G_).

Devices	A	B	C	D	E
Total gate width (μm)	60	120	250	500	2500
Single W_G_ (μm)	60	120	250	500	2500
# of fingers	1	1	1	1	1
Active area (μm^2^)	4560	9120	19,000	38,000	190,000
I_DS,on_ (mA/mm)	73.39	69.735	67.608	46.012	24.875
I_DS,on_ (mA/μm^2^)	9.7 × 10^−^^4^	9.2 × 10^−^^4^	8.8 × 10^−^^4^	6.1 × 10^−^^4^	3.2 × 10^−^^4^

**Table 2:** Design parameters for W_G_ = 60 μm devices with different number of fingers.

Devices	F	G	H	I
Total gate width (μm)	240	600	2400	3600
Single W_G_ (μm)	60	60	60	60
# of fingers	4	10	40	60
Active area (μm^2^)	11,760	36,160	98,160	146,160
I_DS,on_ (mA/mm)	99.87	93.29	120.7	140.56
I_DS,on_ (mA/μm^2^)	5.1 × 10^−^^4^	2.1 × 10^−^^4^	7.4 × 10^−^^5^	5.8 × 10^−^^5^
